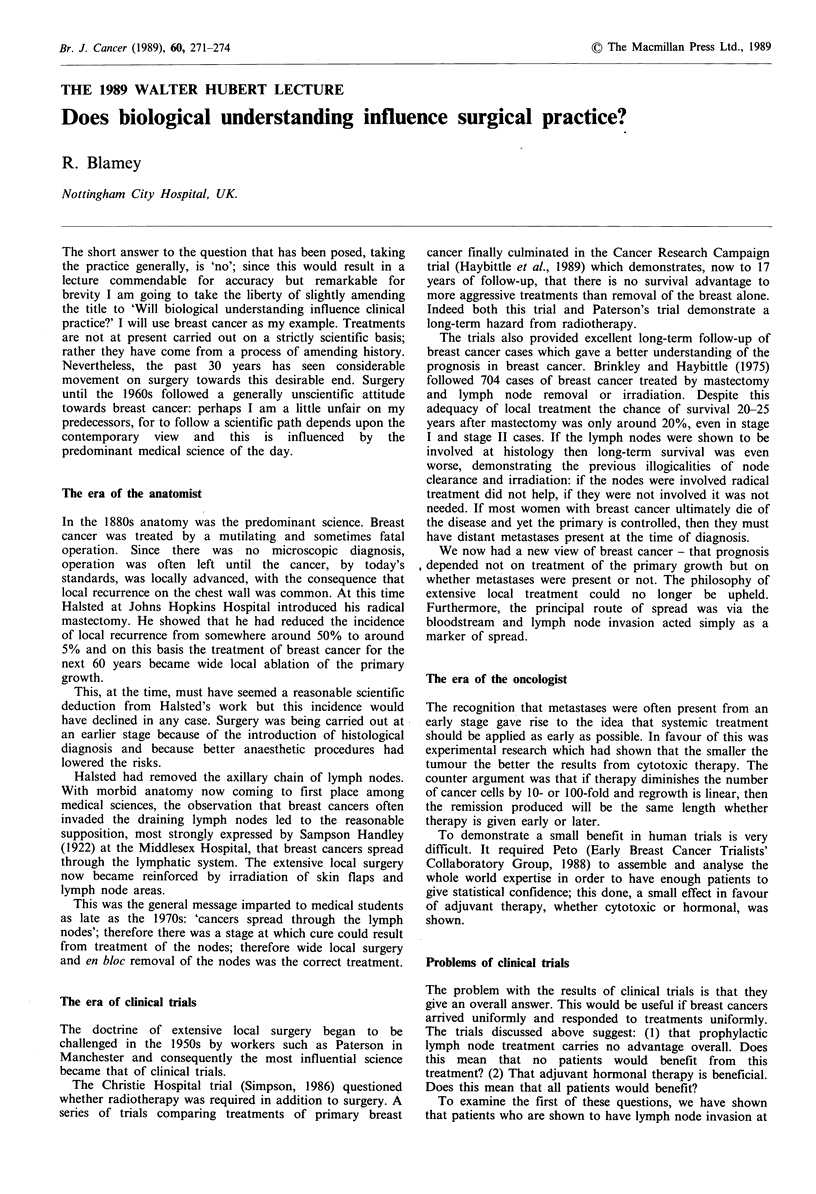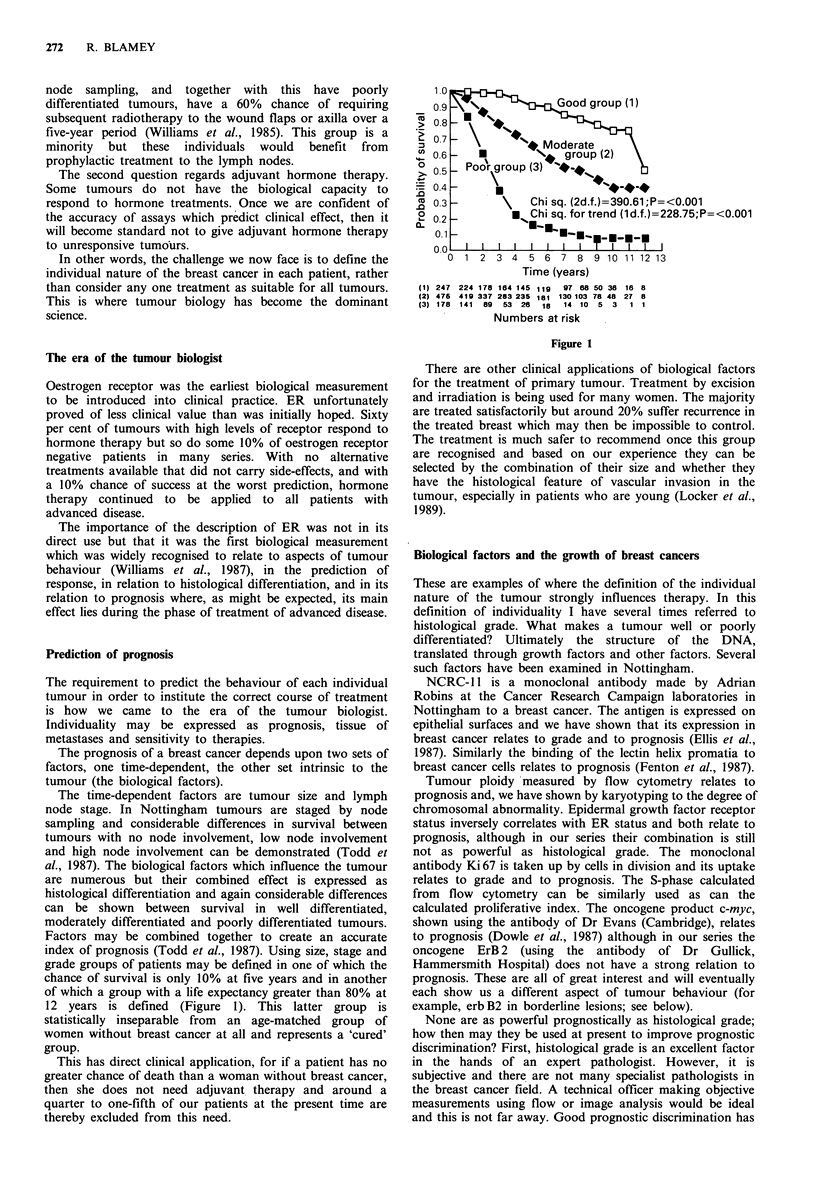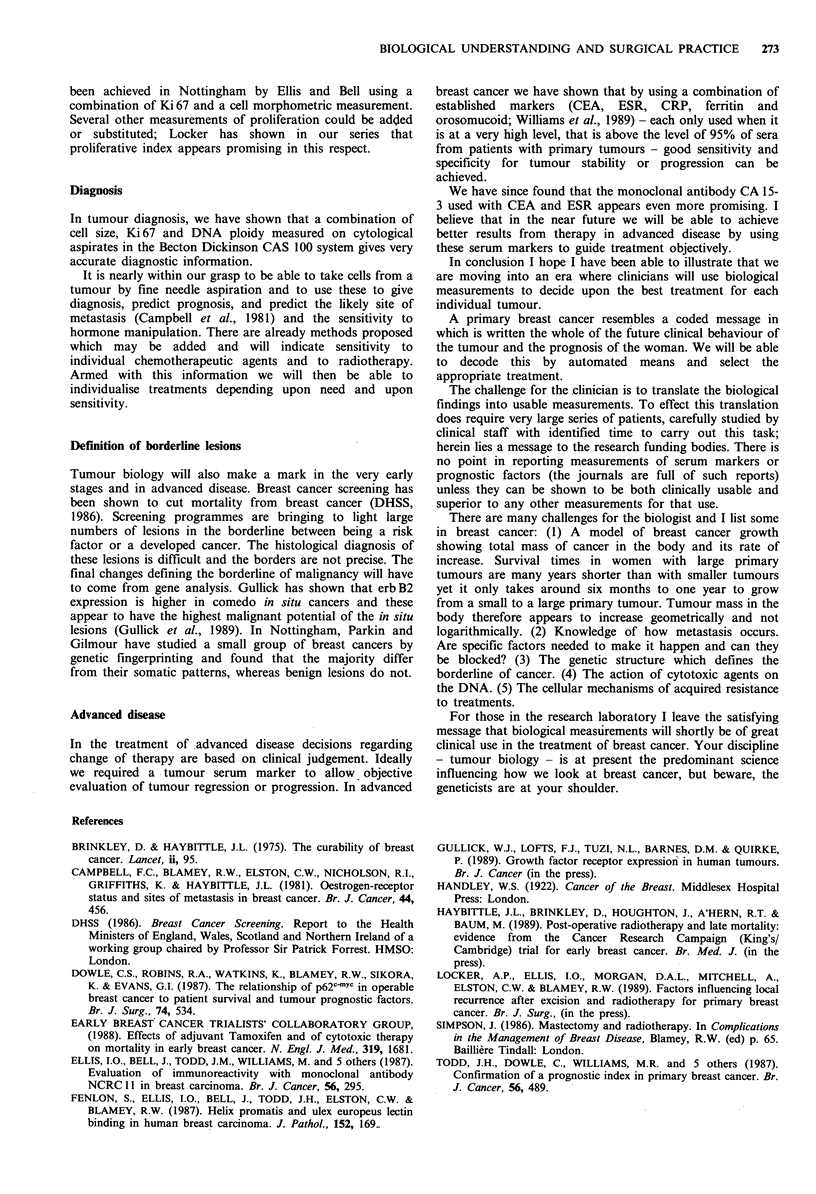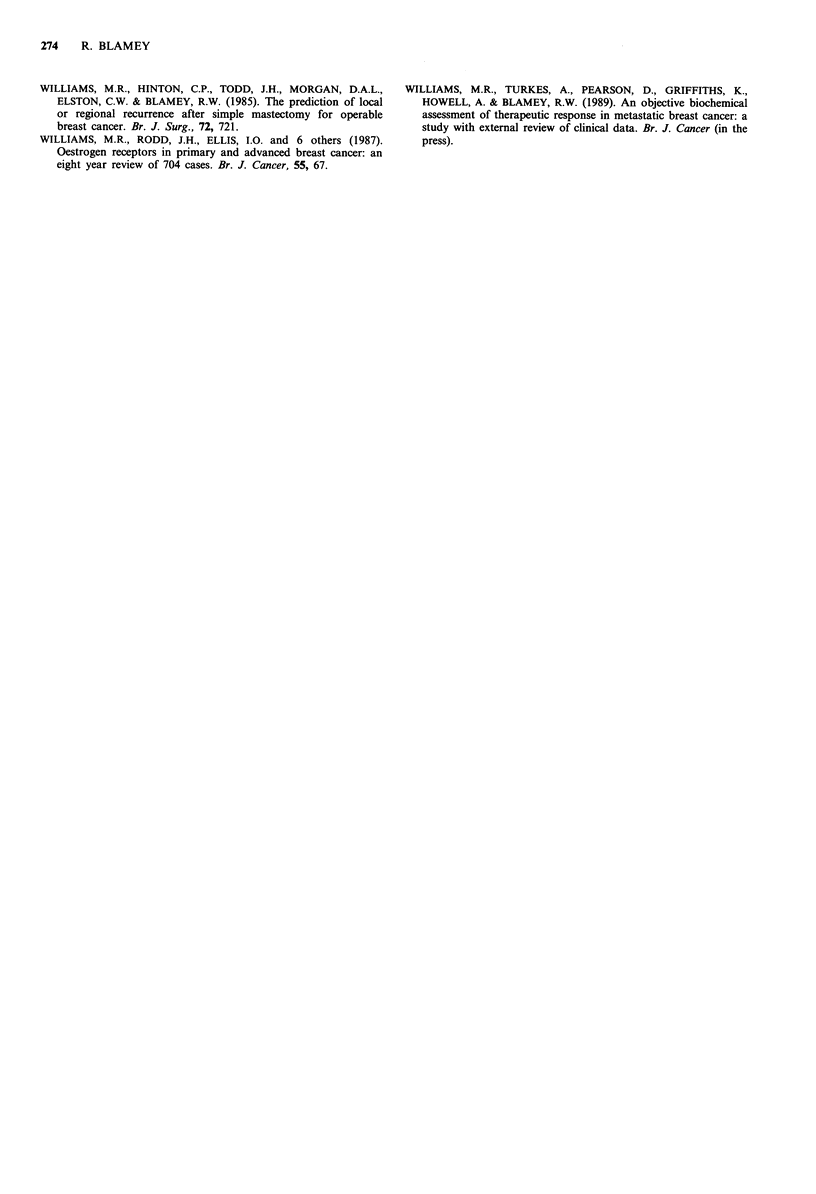# The 1989 Walter Hubert lecture. Does biological understanding influence surgical practice?

**DOI:** 10.1038/bjc.1989.269

**Published:** 1989-09

**Authors:** R. Blamey

**Affiliations:** Nottingham City Hospital, UK.


Br. J. Cancer (1989), 60, 271–274 © The Macmillan Press Ltd., 1989

THE 1989 WALTER HUBERT LECTURE

### Does biological understanding influence surgical practice?

R. Blamey

*Nottingham City Hospital, UK.*

The short answer to the question that has been posed, taking the practice generally, is 'no'; since this would result in a lecture commendable for accuracy but remarkable for brevity I am going to take the liberty of slightly amending the title to 'Will biological understanding influence clinical practice?' I will use breast cancer as my example. Treatments are not at present carried out on a strictly scientific basis; rather they have come from a process of amending history. Nevertheless, the past 30 years has seen considerable movement on surgery towards this desirable end. Surgery until the 1960s followed a generally unscientific attitude towards breast cancer: perhaps I am a little unfair on my predecessors, for to follow a scientific path depends upon the contemporary view and this is influenced by the predominant medical science of the day.

### The era of the anatomist

In the 1880s anatomy was the predominant science. Breast cancer was treated by a mutilating and sometimes fatal operation. Since there was no microscopic diagnosis, operation was often left until the cancer, by today's standards, was locally advanced, with the consequence that local recurrence on the chest wall was common. At this time Halsted at Johns Hopkins Hospital introduced his radical mastectomy. He showed that he had reduced the incidence of local recurrence from somewhere around 50% to around 5% and on this basis the treatment of breast cancer for the next 60 years became wide local ablation of the primary growth.

This, at the time, must have seemed a reasonable scientific deduction from Halsted's work but this incidence would have declined in any case. Surgery was being carried out at an earlier stage because of the introduction of histological diagnosis and because better anaesthetic procedures had lowered the risks.

Halsted had removed the axillary chain of lymph nodes. With morbid anatomy now coming to first place among medical sciences, the observation that breast cancers often invaded the draining lymph nodes led to the reasonable supposition, most strongly expressed by Sampson Handley (1922) at the Middlesex Hospital, that breast cancers spread through the lymphatic system. The extensive local surgery now became reinforced by irradiation of skin flaps and lymph node areas.

This was the general message imparted to medical students as late as the 1970s: 'cancers spread through the lymph nodes'; therefore there was a stage at which cure could result from treatment of the nodes; therefore wide local surgery and *en bloc* removal of the nodes was the correct treatment.

### The era of clinical trials

The doctrine of extensive local surgery began to be challenged in the 1950s by workers such as Paterson in Manchester and consequently the most influential science became that of clinical trials.

The Christie Hospital trial (Simpson, 1986) questioned whether radiotherapy was required in addition to surgery. A series of trials comparing treatments of primary breast cancer finally culminated in the Cancer Research Campaign trial (Haybittle *et al*., 1989) which demonstrates, now to 17 years of follow-up, that there is no survival advantage to more aggressive treatments than removal of the breast alone. Indeed both this trial and Paterson's trial demonstrate a long-term hazard from radiotherapy.

The trials also provided excellent long-term follow-up of breast cancer cases which gave a better understanding of the prognosis in breast cancer. Brinkley and Haybittle (1975) followed 704 cases of breast cancer treated by mastectomy and lymph node removal or irradiation. Despite this adequacy of local treatment the chance of survival 20–25 years after mastectomy was only around 20%, even in stage I and stage II cases. If the lymph nodes were shown to be involved at histology then long-term survival was even worse, demonstrating the previous illogicalities of node clearance and irradiation: if the nodes were involved radical treatment did not help, if they were not involved it was not needed. If most women with breast cancer ultimately die of the disease and yet the primary is controlled, then they must have distant metastases present at the time of diagnosis.

We now had a new view of breast cancer – that prognosis depended not on treatment of the primary growth but on whether metastases were present or not. The philosophy of extensive local treatment could no longer be upheld. Furthermore, the principal route of spread was via the bloodstream and lymph node invasion acted simply as a marker of spread.

### The era of the oncologist

The recognition that metastases were often present from an early stage gave rise to the idea that systemic treatment should be applied as early as possible. In favour of this was experimental research which had shown that the smaller the tumour the better the results from cytotoxic therapy. The counter argument was that if therapy diminishes the number of cancer cells by 10- or 100-fold and regrowth is linear, then the remission produced will be the same length whether therapy is given early or later.

To demonstrate a small benefit in human trials is very difficult. It required Peto (Early Breast Cancer Trialists' Collaboratory Group, 1988) to assemble and analyse the whole world expertise in order to have enough patients to give statistical confidence; this done, a small effect in favour of adjuvant therapy, whether cytotoxic or hormonal, was shown.

### Problems of clinical trials

The problem with the results of clinical trials is that they give an overall answer. This would be useful if breast cancers arrived uniformly and responded to treatments uniformly. The trials discussed above suggest: (1) that prophylactic lymph node treatment carries no advantage overall. Does this mean that no patients would benefit from this treatment? (2) That adjuvant hormonal therapy is beneficial. Does this mean that all patients would benefit?

To examine the first of these questions, we have shown that patients who are shown to have lymph node invasion at node sampling, and together with this have poorly differentiated tumours, have a 60% chance of requiring subsequent radiotherapy to the wound flaps or axilla over a five-year period (Williams *et al.*, 1985). This group is a minority but these individuals would benefit from prophylactic treatment to the lymph nodes.

The second question regards adjuvant hormone therapy. Some tumours do not have the biological capacity to respond to hormone treatments. Once we are confident of the accuracy of assays which predict clinical effect, then it will become standard not to give adjuvant hormone therapy to unresponsive tumours.

In other words, the challenge we now face is to define the individual nature of the breast cancer in each patient, rather than consider any one treatment as suitable for all tumours. This is where tumour biology has become the dominant science.

### The era of the tumour biologist

Oestrogen receptor was the earliest biological measurement to be introduced into clinical practice. ER unfortunately proved of less clinical value than was initially hoped. Sixty per cent of tumours with high levels of receptor respond to hormone therapy but so do some 10% of oestrogen receptor negative patients in many series. With no alternative treatments available that did not carry side-effects, and with a 10% chance of success at the worst prediction, hormone therapy continued to be applied to all patients with advanced disease.

The importance of the description of ER was not in its direct use but that it was the first biological measurement which was widely recognised to relate to aspects of tumour behaviour (Williams *et al.*, 1987), in the prediction of response, in relation to histological differentiation, and in its relation to prognosis where, as might be expected, its main effect lies during the phase of treatment of advanced disease.

### Prediction of prognosis

The requirement to predict the behaviour of each individual tumour in order to institute the correct course of treatment is how we came to the era of the tumour biologist. Individuality may be expressed as prognosis, tissue of metastases and sensitivity to therapies.

The prognosis of a breast cancer depends upon two sets of factors, one time-dependent, the other set intrinsic to the tumour (the biological factors).

The time-dependent factors are tumour size and lymph node stage. In Nottingham tumours are staged by node sampling and considerable differences in survival between tumours with no node involvement, low node involvement and high node involvement can be demonstrated (Todd *et al.*, 1987). The biological factors which influence the tumour are numerous but their combined effect is expressed as histological differentiation and again considerable differences can be shown between survival in well differentiated, moderately differentiated and poorly differentiated tumours. Factors may be combined together to create an accurate index of prognosis (Todd *et al.*, 1987). Using size, stage and grade groups of patients may be defined in one of which the chance of survival is only 10% at five years and in another of which a group with a life expectancy greater than 80% at 12 years is defined (Figure 1). This latter group is statistically inseparable from an age-matched group of women without breast cancer at all and represents a 'cured' group.

This has direct clinical application, for if a patient has no greater chance of death than a woman without breast cancer, then she does not need adjuvant therapy and around a quarter to one-fifth of our patients at the present time are thereby excluded from this need.

**Figure 1**

There are other clinical applications of biological factors for the treatment of primary tumour. Treatment by excision and irradiation is being used for many women. The majority are treated satisfactorily but around 20% suffer recurrence in the treated breast which may then be impossible to control. The treatment is much safer to recommend once this group are recognised and based on our experience they can be selected by the combination of their size and whether they have the histological feature of vascular invasion in the tumour, especially in patients who are young (Locker *et al.*, 1989).

### Biological factors and the growth of breast cancers

These are examples of where the definition of the individual nature of the tumour strongly influences therapy. In this definition of individuality I have several times referred to histological grade. What makes a tumour well or poorly differentiated? Ultimately the structure of the DNA, translated through growth factors and other factors. Several such factors have been examined in Nottingham.

NCRC-11 is a monoclonal antibody made by Adrian Robins at the Cancer Research Campaign laboratories in Nottingham to a breast cancer. The antigen is expressed on epithelial surfaces and we have shown that its expression in breast cancer relates to grade and to prognosis (Ellis *et al.*, 1987). Similarly the binding of the lectin helix promatia to breast cancer cells relates to prognosis (Fenton *et al.*, 1987).

Tumour ploidy measured by flow cytometry relates to prognosis and, we have shown by karyotyping to the degree of chromosomal abnormality. Epidermal growth factor receptor status inversely correlates with ER status and both relate to prognosis, although in our series their combination is still not as powerful as histological grade. The monoclonal antibody Ki 67 is taken up by cells in division and its uptake relates to grade and to prognosis. The S-phase calculated from flow cytometry can be similarly used as can the calculated proliferative index. The oncogene product c-*myc*, shown using the antibody of Dr Evans (Cambridge), relates to prognosis (Dowle *et al.*, 1987) although in our series the oncogene ErB 2 (using the antibody of Dr Gullick, Hammersmith Hospital) does not have a strong relation to prognosis. These are all of great interest and will eventually each show us a different aspect of tumour behaviour (for example, erb B2 in borderline lesions; see below).

None are as powerful prognostically as histological grade; how then may they be used at present to improve prognostic discrimination? First, histological grade is an excellent factor in the hands of an expert pathologist. However, it is subjective and there are not many specialist pathologists in the breast cancer field. A technical officer making objective measurements using flow or image analysis would be ideal and this is not far away. Good prognostic discrimination has been achieved in Nottingham by Ellis and Bell using a combination of Ki 67 and a cell morphometric measurement. Several other measurements of proliferation could be added or substituted; Locker has shown in our series that proliferative index appears promising in this respect.

### Diagnosis

In tumour diagnosis, we have shown that a combination of cell size, Ki 67 and DNA ploidy measured on cytological aspirates in the Becton Dickinson CAS 100 system gives very accurate diagnostic information.

It is nearly within our grasp to be able to take cells from a tumour by fine needle aspiration and to use these to give diagnosis, predict prognosis, and predict the likely site of metastasis (Campbell *et al.*, 1981) and the sensitivity to hormone manipulation. There are already methods proposed which may be added and will indicate sensitivity to individual chemotherapeutic agents and to radiotherapy. Armed with this information we will then be able to individualise treatments depending upon need and upon sensitivity.

### Definition of borderline lesions

Tumour biology will also make a mark in the very early stages and in advanced disease. Breast cancer screening has been shown to cut mortality from breast cancer (DHSS, 1986). Screening programmes are bringing to light large numbers of lesions in the borderline between being a risk factor or a developed cancer. The histological diagnosis of these lesions is difficult and the borders are not precise. The final changes defining the borderline of malignancy will have to come from gene analysis. Gullick has shown that erb B2 expression is higher in comedo *in situ* cancers and these appear to have the highest malignant potential of the *in situ* lesions (Gullick *et al.*, 1989). In Nottingham, Parkin and Gilmour have studied a small group of breast cancers by genetic fingerprinting and found that the majority differ from their somatic patterns, whereas benign lesions do not.

### Advanced disease

In the treatment of advanced disease decisions regarding change of therapy are based on clinical judgement. Ideally we required a tumour serum marker to allow objective evaluation of tumour regression or progression. In advanced breast cancer we have shown that by using a combination of established markers (CEA, ESR, CRP, ferritin and orosomucoid; Williams *et al.*, 1989) – each only used when it is at a very high level, that is above the level of 95% of sera from patients with primary tumours – good sensitivity and specificity for tumour stability or progression can be achieved.

We have since found that the monoclonal antibody CA 15-3 used with CEA and ESR appears even more promising. I believe that in the near future we will be able to achieve better results from therapy in advanced disease by using these serum markers to guide treatment objectively.

In conclusion I hope I have been able to illustrate that we are moving into an era where clinicians will use biological measurements to decide upon the best treatment for each individual tumour.

A primary breast cancer resembles a coded message in which is written the whole of the future clinical behaviour of the tumour and the prognosis of the woman. We will be able to decode this by automated means and select the appropriate treatment.

The challenge for the clinician is to translate the biological findings into usable measurements. To effect this translation does require very large series of patients, carefully studied by clinical staff with identified time to carry out this task; herein lies a message to the research funding bodies. There is no point in reporting measurements of serum markers or prognostic factors (the journals are full of such reports) unless they can be shown to be both clinically usable and superior to any other measurements for that use.

There are many challenges for the biologist and I list some in breast cancer: (1) A model of breast cancer growth showing total mass of cancer in the body and its rate of increase. Survival times in women with large primary tumours are many years shorter than with smaller tumours yet it only takes around six months to one year to grow from a small to a large primary tumour. Tumour mass in the body therefore appears to increase geometrically and not logarithmically. (2) Knowledge of how metastasis occurs. Are specific factors needed to make it happen and can they be blocked? (3) The genetic structure which defines the borderline of cancer. (4) The action of cytotoxic agents on the DNA. (5) The cellular mechanisms of acquired resistance to treatments.

For those in the research laboratory I leave the satisfying message that biological measurements will shortly be of great clinical use in the treatment of breast cancer. Your discipline – tumour biology – is at present the predominant science influencing how we look at breast cancer, but beware, the geneticists are at your shoulder.

### References

BRINKLEY, D. & HAYBITTLE, J.L. (1975). The curability of breast cancer. *Lancet*, **ii**, 95.

CAMPBELL, F.C., BLAMEY, R.W., ELSTON, C.W., NICHOLSON, R.I., GRIFFITHS, K. & HAYBITTLE, J.L. (1981). Oestrogen-receptor status and sites of metastasis in breast cancer. *Br. J. Cancer*, **44**, 456.

DHSS (1986). *Breast Cancer Screening*. Report to the Health Ministers of England, Wales, Scotland and Northern Ireland of a working group chaired by Professor Sir Patrick Forrest. HMSO: London.

DOWLE, C.S., ROBINS, R.A., WATKINS, K., BLAMEY, R.W., SIKORA, K. & EVANS, G.I. (1987). The relationship of $p62^{c\text{-}myc}$ in operable breast cancer to patient survival and tumour prognostic factors. *Br. J. Surg.*, **74**, 534.

EARLY BREAST CANCER TRIALISTS' COLLABORATORY GROUP, (1988). Effects of adjuvant Tamoxifen and of cytotoxic therapy on mortality in early breast cancer. *N. Engl. J. Med.*, **319**, 1681.

ELLIS, I.O., BELL, J., TODD, J.M., WILLIAMS, M. and 5 others (1987). Evaluation of immunoreactivity with monoclonal antibody NCRC 11 in breast carcinoma. *Br. J. Cancer*, **56**, 295.

FENLON, S., ELLIS, I.O., BELL, J., TODD, J.H., ELSTON, C.W. & BLAMEY, R.W. (1987). Helix promatis and ulex europeus lectin binding in human breast carcinoma. *J. Pathol.*, **152**, 169.

GULLICK, W.J., LOFTS, F.J., TUZI, N.L., BARNES, D.M. & QUIRKE, P. (1989). Growth factor receptor expression in human tumours. *Br. J. Cancer* (in the press).

HANDLEY, W.S. (1922). *Cancer of the Breast*. Middlesex Hospital Press: London.

HAYBITTLE, J.L., BRINKLEY, D., HOUGHTON, J., A'HERN, R.T. & BAUM, M. (1989). Post-operative radiotherapy and late mortality: evidence from the Cancer Research Campaign (King's/Cambridge) trial for early breast cancer. *Br. Med. J.* (in the press).

LOCKER, A.P., ELLIS, I.O., MORGAN, D.A.L., MITCHELL, A., ELSTON, C.W. & BLAMEY, R.W. (1989). Factors influencing local recurrence after excision and radiotherapy for primary breast cancer. *Br. J. Surg.*, (in the press).

SIMPSON, J. (1986). Mastectomy and radiotherapy. In *Complications in the Management of Breast Disease*, Blamey, R.W. (ed) p. 65. Baillière Tindall: London.

TODD, J.H., DOWLE, C., WILLIAMS, M.R. and 5 others (1987). Confirmation of a prognostic index in primary breast cancer. *Br. J. Cancer*, **56**, 489.

WILLIAMS, M.R., HINTON, C.P., TODD, J.H., MORGAN, D.A.L., ELSTON, C.W. & BLAMEY, R.W. (1985). The prediction of local or regional recurrence after simple mastectomy for operable breast cancer. *Br. J. Surg.*, **72**, 721.

WILLIAMS, M.R., RODD, J.H., ELLIS, I.O. and 6 others (1987). Oestrogen receptors in primary and advanced breast cancer: an eight year review of 704 cases. *Br. J. Cancer*, **55**, 67.

WILLIAMS, M.R., TURKES, A., PEARSON, D., GRIFFITHS, K., HOWELL, A. & BLAMEY, R.W. (1989). An objective biochemical assessment of therapeutic response in metastatic breast cancer: a study with external review of clinical data. *Br. J. Cancer* (in the press).